# A Fragmented Mind: Altered States of Consciousness and Spirit Possession Between Rituals and Therapy

**DOI:** 10.1007/s12124-025-09929-0

**Published:** 2025-07-30

**Authors:** Donato Giuseppe Leo

**Affiliations:** Department of Pharmacology and Therapeutics, Institute of Systems, Molecular and Integrative Biology, Faculty of Health and Life Sciences, University of Liverpool, Liverpool, UK

**Keywords:** Altered states of consciousness, Consciousness, Exorcism, Spirit possession, Trance states

## Abstract

This paper focuses on understanding how cultural influences, social expectancy, and personal beliefs shape the perception of altered states of consciousness and how these mental states have been interpreted as a way to communicate with the spiritual world. Altered states of consciousness are commonly encountered in religious, spiritual, and therapeutic (e.g., hypnosis) practices. While neurophysiological aspects of altered states of consciousness are an important part of understanding the nature of human consciousness, the cultural meaning that these states of mind assume in different communities is equally fundamental. The phenomenon of spirit possession is a meaningful example of how sociocultural factors influence and shape the perception of altered states of consciousness. An understanding of the meaning of spirit possession as a tool to “exorcise” individual trauma or to address communal fears and turmoil is provided here. From the historical concept of the supernatural nature of physical and mental illness through the discussion of rituals aiming at casting out or taming the possessing spirit, this paper wants to provide an understanding of how sociocultural factors have been determinant in embedding altered states of consciousness in religious and spiritual practices, and how these states are of therapeutic value for mental wellbeing.

## Introduction

Altered states of consciousness (ASCs) refer to any mental state different from normal waking consciousness (Vaitl et al., 2013). They are characterised by changes in awareness, cognition, sense of self and perception (e.g., daydreaming, trance states) (Vaitl et al., 2013). ASCs do often involve dissociation (disconnection of some aspects of mental functioning from conscious awareness, such as perception, sense of identity, memory, or emotions) (Lanius, 2015) and can be induced voluntarily or involuntarily using psychedelic drugs (e.g., LSD), chanting, dancing, meditation, praying, or hypnosis (Preller & Vollenweider, 2018; Vaitl et al., 2013). ASCs such as trance states are commonly encountered in religious (e.g., spirit possession, religious ecstasy) (Lewis, 2002), spiritual (e.g., meditation) (Lutz et al., 2006), and therapeutic (e.g., hypnosis) (Leo et al., 2024) contexts. ASCs have gained symbolic (cultural) value in cultures all around the world, leading to the adoption of spiritual and religious rituals aiming at inducing alteration of normal consciousness as a means to communicate with the supernatural (Price-Williams & Hughes, 1994). A meaningful example of how sociocultural factors shape the perception of ASCs can be encountered in the phenomenon of spirit possession.

Spirit possession is the belief that a supernatural entity can take over the control of someone’s body and mind, influencing their actions and behaviours (Cohen, 2008). Although in some spiritual practices (e.g., Shamanism) spirit possession may be seen as beneficial for the individual (e.g., granting them healing powers) (Lewis, 2002), in some religious traditions (e.g., Christianity) it may be seen as negative (e.g., cases of demonic possession) (Cook, 2025). While medicine discuss allegedly symptoms of spirit possession as a matter of mental and physical illness (e.g., neurological – epilepsy; or psychiatric – schizophrenia) (Perrotta, 2019), religion and cultural traditions have elaborated complex rituals of symbolic value to “exorcise” (i.e., cast out) the spiritual entity from the possessed individual(Young, 2016) or to integrate it with the individual (“adorcism”), often as a spiritual guide and companion (Lewis, 2002). These rituals aim to induce a trance state (e.g., through chanting, dancing, or praying), where the individual is free to exhibit the stereotyped behaviour associated with their state of being possessed, reaching mystic ecstasy that leads to spiritual and physical healing (Lewis, 2002). Although in several social, religious, and cultural contexts spirit possession has been a way to label mental and physical issues (Cavanna et al., 2010; Ward, 1980), with rituals aiming to ‘evict the demon’ considering the cause of the symptoms; in other contexts, spirit possession has been seen as a source of divine intervention and guidance (Kawamura, 2003). The religious and spiritual perception of physical and mental disorders has always sought a cause of supernatural origin, especially in the case of psychosomatic symptoms, delusions, and seizures, which have often been seen as of a divine or diabolical nature (Hecker et al., 2016). Moreover, mental health and psychiatric disorders have always been hidden behind the fear of stigma and social judgment, especially in religious communities (Lloyd & Panagopoulos, 2022), and a form of psychological support has been delivered in many instances in the form of religious or spiritual interventions (Nooney & Woodrum, 2002). Based on cultural significance, communities have seen in ASCs a means to foresee the future, cure illnesses, and reach unity with the divine, as well as the cause of disease, the work of evil spirits, and the ruin of the soul. ASCs provide a window into understanding human consciousness and the mind-body connection. Consciousness is not only a biological phenomenon, but is also shaped by culture, social contexts, and beliefs. Thus, integrating physiological mechanisms with social and cultural factors is essential in understanding ASCs and their symbolic and therapeutic value. Spirit possessions are a relevant example of culturally influenced integration of ASCs, which has been carefully investigated from an anthropological, psychological, and medical point of view. Therefore, the aims of this paper are to discuss: (i) how sociocultural influences have integrated ASCs in the sphere of spirit possession, and (ii) the religious and spiritual integration of ASCs in practices aiming at emotional and mental healing.

### A Devilish Illness: Physical and Mental Disease Across Time and Cultures

A brief overview of how mankind has understood and treated diseases through the centuries is essential to grasp the role and meaning of spirit possession, especially in physical and mental health. The animistic approach of ancient civilisations toward nature, and the role of the supernatural in everyday life, has been essential in the development of magic as a mean to influence nature and reality (as described by Freud in his idea of the omnipotence of thoughts) (Freud, 2012), thus supporting its practice in ancient medical treatments. Ancient medicine relied on a combination of religious, magical, and naturalistic approaches (e.g., herbal remedies) (Nutton, 2012). Diagnosis of disease and prescription of specific cures were already well documented by the ancient Egyptians and the Sumerians, who tried to understand the cause of disease and illness, often providing supernatural and religious explanations (Heeßel, 2004; Karenberg & Leitz, 2001; Walker, 1990). Sumerians thought of illness as a punishment for sins against the gods or caused by the attack of evil spirits (Heeßel, 2004; Retief & Cilliers, 2007). Thus, magic spells and amulets, together with exorcisms to cast out the evil spirits, were essential to assist the recovery of the individual affected by the disease (Heeßel, 2004; Retief & Cilliers, 2007). Similarly, for the ancient Egyptians, diseases were associated with evil spirits and spirit possession, but also induced by natural causes (Zucconi, 2007). To treat the disease, physicians adopted practices that embedded magic rituals, exorcisms, medical procedures, and medical prescriptions (Karenberg & Leitz, 2001; Walker, 1990). In ancient Greece, medicine was initially based on religious rites administered by priests at the temples dedicated to the worship of the healer-god Asclepios (Savel & Munro, 2014). For them, illness was caused by supernatural phenomena. It was Hippocrates (460 − 370 BC) who later on proposed that diseases were caused by natural phenomena instead, separating medicine from religion and leading the way to modern medicine (Jacques Jouanna & Van der Eijk, 2012). Advancements to a more scientific approach to medicine were made with Galen (129–216 CE), who strongly contributed to the development of anatomy, physiology, neurology, pharmacology, and pathology (Jacques Jouanna & Van der Eijk, 2012).

The Jewish historian Flavius Josephus (c.37–100 CE), in his ‘*Antiquities of the Jews’* report the practice of Jewish exorcism tradition being attributed to King Solomon, also reporting a demonstration of exorcism he claimed to be witnessed (where the exorcist would use a ring and root to drawn the demon out of the nostrils of the possessed) (Josephus, 2006), showing how the concepts of spirit possession and exorcism were relevant also in Jewish tradition. Examples of exorcism as a therapeutic tool can also be seen in the New Testament. Jesus is depicted in several instances practising exorcisms on individuals who were presented as being “possessed”, manifesting tracts that can now be associated with physical and mental illness (Cook & Hamley, 2020). Famous is in this regard the exorcism performed by Jesus on the man possessed by a multitude (a “legion”) of evil spirits reported in the story of the “Gerasene demonic” of the New Testament (Matthew 8:28, Luke 8:26–39, and Mark 5:1–20) (*The Holy Bible: New King James Version.*, 1982), describing the unusual behaviour of the possessed man (not wearing clothes and living in a tomb – as probably exiled by the community for his unusual behaviour).

The perception of physical or mental diseases as of supernatural nature is still largely accepted in some communities (Mathison et al., 1895; Simba et al., 2023), with religious beliefs of miraculous cures still present all around the world (Leal et al., 2024). People with mental disorders often suffer high levels of stigma, and the disorders are commonly associated with supernatural causes, such as spirit possession, in several religious communities (Kate et al., 2012). In some cases, instead, mental illness is seen as the cause of spiritual weakening that can make the individual more susceptible to demonic possession (Lloyd & Panagopoulos, 2023). This reinforces the stigma about mental illness present in many communities, with religious individuals often refusing or delaying medical diagnosis and treatment in favour of alternative interventions (such as prayers and religious rituals) (Mathison et al., 1895). Religious and personal beliefs can also negatively affect preventive strategies, such as vaccine campaigns (Tiwana & Smith, 2024). Moreover, access to life-saving medical procedures, such as blood transfusion, can be strongly obstructed by religious beliefs (Feane & Uprichard, 2025). On the other hand, religious beliefs and behaviours can also support mental health, alleviating one’s negative emotions or life stressful events (religious coping) (Pargament, 2001). However, while positive religious coping (e.g., spiritual connection) has demonstrated positive outcomes on mental wellbeing and self-esteem (Ano & Vasconcelles, 2005), negative religious coping (e.g., demonic reappraisal) has been demonstrated to lead to more significant emotional distress (such as anxiety and depression) (Ano & Vasconcelles, 2005). Nevertheless, in some instances, negative religious coping can still lead to positive outcomes (such as spiritual growth and increased self-esteem), as it may represent spiritual struggles that are a pathway to spiritual growth (Ano & Vasconcelles, 2005).

### Voices in My Mind: Dissociation, Altered States of Consciousness, and Spirit Possession

Despite the nature of spirit possession being strongly related to the social and cultural context where it manifests, often as an explanation for physical and mental illness, it is also important to note that this phenomenon can also be experienced by people who do not necessarily present symptoms of mental illness. Indeed, natural neurophysiological and psychophysiological changes can lead to experience ASCs, inducing a state of mind where the perception of the situation by the possessed individual becomes embedded in sociocultural and personal beliefs, thus manifesting as the idea of supernatural work. To understand this concept is essential to understand what dissociation is, and its role in inducing ASCs (either of pathological or non-pathological nature). Dissociation is a disruption in one or more aspects of psychological functioning, such as consciousness, memories, feelings, and sense of identity (Krause-Utz et al., 2017; Spiegel et al., 2011). Dissociative episodes of various levels naturally occur during the day (e.g., mind-wandering, daydreaming), with individuals more inclined to fantasy-proneness, absorption, and cognitive failure, having a higher tendency to dissociate (Merckelbach et al., 1999). However, severe episodes of dissociation are also characteristic of mental disorders (e.g., dissociative disorders, functional neurological disorders – FNDs) (Espay et al., 2018; Spiegel et al., 2011). The alteration in the individual’s sense of reality and the continuity of their consciousness, typical of dissociation, is a core component of ASCs, both in natural and trauma-related ASCs (Butler, 2006; Lanius, 2015).

Neurophysiological and psychophysiological changes associated with ASCs and dissociation are linked to alterations in brain activity that impact cognitive functions. These changes explain some of the mechanisms behind the “symptoms” and manifestations of spirit possession (e.g., post-possession amnesia or the feeling of being controlled by an external force). More in details, three specific large-scale brain networks in particular (the triple network model of psychopathology) (Menon, 2011) are relevant in the context of ASCs: (i) the Default Mode Network (DMN – involved in self-referential processes, autobiographical memory, and future planning) (Andrews-Hanna et al., 2014), (ii) the Executive Control Network (ECN – regulating goal-oriented behaviour, cognitive control, and attention) (Shen et al., 2020), and (iii) the Salience Network (SN – involved in emotional control, also regulating the switch between self-referential processes and goal oriented thinking) (Goulden et al., 2014). In ASCs, such as mind wandering and daydreaming, it has been observed an increase in self-referential thinking (highlighting increased activity of the DMN) and a reduced focus on external tasks (denoting a decrease in the activity of the ECN) (Zhou & Lei, 2018). Prolonged periods of mind wandering have been associated with low activity of the SN (Bonnelle et al., 2012; Bozhilova et al., 2018). During ASCs, such as hypnosis, a reduced activity of the DMN (Deeley et al., 2012), together with changes in functional connectivity between the DMN, the ECN, and the SN have been observed (Jiang et al., 2017). The reduced functional connectivity between the DMN and ECN observed during ASCs explains the increased suggestibility typical of these states (Jiang et al., 2017), which in turn favours the immersion into a role-play type of setting, where personal beliefs and cultural expectations dictate stereotypical behaviours of how an individual possessed by a spirit should behave. During pathological dissociation, symptoms of depersonalisation and derealisation have been associated with hyperconnectivity within regions of the ECN during the resting state and altered connectivity between the ECN and the DMN (Lebois et al., 2022). This momentary loss of self-identity and contact with reality contributes to the feeling of being controlled by a supernatural entity. Alterations in memory recall and emotional control observed in patients with dissociative disorders have been linked to reduced hippocampus and amygdala volumes (Vermetten et al., 2006). Dissociative amnesia has been observed in both pathological dissociation and in normal ASCs (Kihlstrom & Evans, 2014; Staniloiu & Markowitsch, 2014), and can be linked to failure in activating specific areas of the ECN and of the DMN, together with a decreased connectivity between areas of the DMN and of the SN (Taïb et al., 2023). The effects of dissociation and ASCs on memory can explain why people experiencing spirit possession are often unable to recall their experience once they snap out of possession trance. High anxiety levels have also been observed in individuals reporting an increased occurrence of dissociative experiences (dissociative anxiety) (Belli et al., 2017; Lofthouse et al., 2023), which can be partially explained by the increased DMN activity observed during emotional distress (Coutinho et al., 2016; Yuan et al., 2023). Thus, anxious situations can trigger dissociative episodes that can be interpreted as of supernatural origin based on the cultural and personal beliefs of the individual. Dissociation seems to have a higher occurrence in female individuals (Page & Green, 2007; Raynor & Baslet, 2019), similarly to episodes of spirit possession (De Martino & Zinn, 2005; Lewis, 2002; Seligman & Kirmayer, 2008), which can be potentially explained by the higher intra-functional connectivity of the DMN observed in women compared to men (Ficek-Tani et al., 2023; Mak et al., 2017). However, the role that sociocultural factors have in the incidence of spirit possession in women should not be underestimated (Lewis, 2002; Seligman & Kirmayer, 2008).

Both natural and pathological dissociation can be linked to experiences of spirit possession. While in ritualistic possession, ASCs aiming at natural forms of dissociation can be temporary and in line with the objective of the ritual (Lewis, 2002), in cases of persistent spirit possession (occurring spontaneously, and not linked to specific rituals) it may involve pathological dissociation (e.g., dissociative disorders) or other mental conditions (e.g., schizophrenia) (R. Seligman & L. J. Kirmayer, 2008). Dissociative identity disorders (DID) are defined by the *Diagnostic and Statistical Manual of Mental Disorders* (DMS-5) as the alternation of two or more distinct personality states (alters), seen in some cultures as a form of possession by an external entity (American Psychiatric Association, 2022). In DID, dissociative episodes can induce “inter-identity amnesia”, where one alter does not recall the events that occurred while the other alter took control (Morton, 2017). This is similar to post-trance amnesia reported by people experiencing ASCs and spirit possession, which is related to alterations in the intra- and extra-connectivity of the large-scale network previously discussed. In the *International Classification of Disease for Mortality and Morbidity Statistics* (ICD-11-MMS) (World Health Organization, 2019), dissociative disorders where the individual is convinced to be possessed by an external entity fall under the name of “possession trance disorder”, with the individual manifesting a trance state (not induced by medications or by psychoactive substances) that leads to alteration of consciousness and personal identity, also experiencing the feeling of being controlled by an external possessing entity (sense of involuntariness). The illusion of being controlled by an external agent can be attributed to alteration in self-awareness due to changes in activity and connectivity of the DMN (Jiang et al., 2017; Monsa et al., 2018) experienced during ASCs and spirit possession episodes, together with personal beliefs (Wolfradt, 1997) concerning the possession by a supernatural entity. Moreover, the increased suggestibility related to dissociation (Pekala et al., 2010; Wieder et al., 2022) can lead to culturally-influenced stereotyped behaviours, such as the ones observed during possession rituals. The allegedly “possessed” individual does not necessarily exhibit multiple personalities but can experience other psychosomatic symptoms (conversion symptoms) that fall into the classification of Functional Neurological Disorders (FNDs - previously known as “conversion disorders”, also known as “dissociative neurological symptoms disorders”) (Canna & Seligman, 2020). FNDs involve the experience of neurological symptoms (such as weakness, movement problems, convulsions, and other sensory problems) without a tangible disease affecting the structure of the patient’s body (Aybek & Perez, 2022). Potential causes of FNDs can be stress-related (Hallett et al., 2022) or due to brain dysfunction (Aybek & Vuilleumier, 2016). FNDs’ manifestations and perception can be dictated by sociocultural factors, with social and personal beliefs influencing the perception of the nature of the illness (e.g., of supernatural origin) and the manifestation of the psychosomatic symptoms (Canna & Seligman, 2020; Kirmayer & Santhanam, 2000).The association of FNDs to episodes of spirit possession can be observed, for example, in the phenomenon of Tarantism, where the bite of the tarantula brings a supernatural illness to the victim, who is then forced to exhausting dancing to exorcise the spirit of the spider (De Martino & Zinn, 2005).

### Dancing With the Spirits: An Overview of the Spiritual and Cultural Value of Altered States of Consciousness

The spiritual and cultural value of ASCs can be observed in social and cultural phenomena such as ecstatic religions (Lewis, 2002), which embraced a variety of cults and religions that were and are largely diffused around the world. From the cult of Dionysus (Mysteries of Dionysus – commonly spread during ancient Greece and the Roman period) to Haitian Voodoo, passing from Tarantism, Christian mysticism, and Shamanism, common tracts can be seen in the use of ASCs to communicate with the divine (Marie et al., 2024; Randal et al., 2018). Trance-induced possessions have been depicted and used in many religious practices, through the use of chanting, prayers, and dancing (Marie et al., 2024). Trance states have the ritualistic value of a means to induce possession (from benevolent spirits). Conversely, their ritualistic use can also become a mean to exorcise (cast out) the evil spirit from the body of the possessed (Charles, 1953).

The idea of spirit possession as the cause of illness and spiritual ruin is central in many communities. As previously discussed, the idea that diseases are caused by evil spirits was common in ancient societies, and it is still largely embraced in several rural communities or specific religious cults. An example of possession as illness which needs to be exorcised through rites of symbolic value, aiming to treat conditions of hysteric origin, can be found in the phenomenon of Tarantism (De Martino & Zinn, 2005). Especially in the 16th and 17th centuries, it was common in Southern Italy (mainly in Puglia) suffer from the “bite of the Tarantula” (the wolf spider), with the poor victims exhibiting hysteric behaviours that needed to be exorcised through music and dance (the “Tarantella” or “Pizzica”) (De Martino & Zinn, 2005). Again, the symbolic value of the tarantula and its bite (probably evolving from real accounts of bites suffers by farmers working in the fields and exposed to the spider during the season of the grain crop), was so strongly shaped by the sociocultural influences of the time that it became the root cause of hysteric manifestations in a crisis that had its origin in the mental turmoil of the victim (De Martino & Zinn, 2005). Similarly, the rite to “exorcise” the “tarantato/a” (the person suffering from tarantism), used symbolic values to allow a safe manifestation of psychomotor symptoms and mitigate the internal turmoil presented by the possessed (De Martino & Zinn, 2005; Lewis, 2002). As highlighted by Lewis (Lewis, 2002) and De Martino (De Martino & Zinn, 2005), the recurrence of the symptoms in concomitance of specific celebrations, could have seen as a socially accepted way for the victim to express their malcontent and rebel to their status (e.g., forced wedding), acknowledging the dissent of the victim by the community while still integrating them in their moral and communal rules.

Even more well-known is the idea of demon possession that is observed in Christianity, with the evil spirit entering the body of the individual, influencing their behaviour and causing physical and spiritual illness. In the 1980s, the Satanic Panic hit the USA, with a multitude of individuals proclaiming to have been victims of allegedly satanic rituals (Cleary, 2022). The following multitude of movies have then increased the spotlight on alleged cases of demonic possession, depicting the role of the Devil in manifesting all sorts of supernatural phenomena (e.g., levitating or psychokinetic powers) surrounding the possessed (Chambers, 2021). These movies have drastically affected the social perception of spirit possession in Western society, leading to an increased report of cases of allegedly demonic possession (Giordan & Possamai, 2016). The hysteric origin (rather than supernatural) of demonic possession (as intended by Christianity) was already observed in the late 18th Century, with the case of Johann Joseph Gassner, famous at the time for his exorcisms and his ability to cure people from the devil (Peter, 2005). It was Franz A. Mesmer (known for his claims on animal magnetism) who in 1775 debunked Gassner’s practice not as a matter of supernatural entity, but as a proof of curative trance (or, accordingly to Mesmer’s ideas, of “animal magnetism”) (Peter, 2005). The people “exorcised” by Gassner were presenting a form of hysteric behaviour (i.e. FNDs) that was manifesting in stereotypical manifestations shaped by the sociocultural background of the “possessed”. The approach to the ritual of exorcism performed by Gassner as integration of the symbolic value of the rite (in line with the sociocultural context of the time) and of early concepts of what we could now consider “psychotherapy”, was successful in reducing the hysteric manifestation of the possessed, exorcising the “devil” within (Peter, 2005).

A more ecstatic approach to spirit possession, where the idea of being possessed is not seen in a negative connotation, but rather as a valuable event necessary to the community, can be seen with the *loa* spirits of Haitian Voodoo (Desmangles, 2000). During the possession (happening during sacred ceremonies that involve drums, songs, and dancing), the *loa* (spirits that serves as intermediate between humans and the creator of the universe) enters their human vessel through the head, displacing one of the two halves of the human souls, causing the individual to convulse (Desmangles, 2000). The possession can last from a few hours to several days, and once the *loa* leaves, the possessed has no memory of what happened during the possession phase (dissociative amnesia) (Desmangles, 2000). The possession facilitates the interaction between *loa* and humans, allowing the spirits to provide admonishments or offer advice and healing powers (Desmangles, 2000). Another example of positive spirit possession can be observed in Christian mysticism, where individuals seeking unity with God accomplish this through contemplation, purification, and prayer, aiming to reach illumination (James, 1902; Underhill, 2002). Similarly, in Pentecostal Christianity, the union with the supernatural (the divine) is seen as beneficial, with possession by the Holy Spirit (like in the Acts of Apostles 2:1–4) (*The Holy Bible: New King James Version.*, 1982) seen as a source of divine guidance (James, 1902; Robbins, 2004). On the contrary, the possession by unholy spirits (demons), usually due to sinful behaviours, is the cause of spiritual ruin and disease (Robbins, 2004; Russell, 1987).

Characteristics of the possession behaviour are strictly correlated to the social, religious, and cultural beliefs on how a possessed individual should behave (Bourguignon, 1976; Goodman, 1988). In Christianity, the possessed person may exhibit aversion toward symbols of Christianity (e.g., the sacred cross, or the Bible), also exhibiting changes in the tone of voice, and blasphemous behaviour (Bourguignon, 1976). In other cultural contexts, such as Haitian Voodoo, the possessed individual is the dispenser of prophecies of upcoming events. In Tarantism, the bite from the Tarantula leads to several symptoms, such as hysteric behaviour, restlessness, depressive mood (melancholia), hallucination, and especially the urge to dance violently (De Martino & Zinn, 2005). The dance, its key characteristic, is done accordingly to the specific tone and instrument (among the tambourine, violin, guitar, and accordion) that resonates with the specific tarantula that bit the individual (De Martino & Zinn, 2005). Even the type of dance (either fast or slow-paced) depended on the type of tarantula from which the bite originated (De Martino & Zinn, 2005).

Spirit possession may indeed embrace a large variety of physical and mental diseases, which in the context of the time were defined as of divine or demonic influence. We have seen that in antiquity and still in some rural communities, illness was and is associated with the influence of evil spirits, as a form of divine punishment, or the work of witchcraft (Kirmayer, 2001). We have then a model of spirit possession that is related to any kind of physical and mental illness that an individual may encounter, being the influence of supernatural entities relevant in preserving or disrupting the health of an individual. Common mental health conditions, such as uncontrollable anxiety and depression, have been attributed in the past to supernatural origins, due to difficulties in explaining the origin of the symptoms that did not seem to come from the observation of physical dysfunction (Kirmayer, 2001). Delusions typical of mental illness, such as schizophrenia, may have been seen as a form of spirit possession, with the affected individual complaining of hearing voices in their head, as well as seen things and beings that were invisible to others (Pietkiewicz et al., 2021). Indeed, auditory allucinations can be interpreted dirrefently by the subject experiencing them based on cultural variations (Luhrmann et al., 2015). A specific role takes the episodes of voluntary and involuntary dissociative episodes (either natural or pathological), as these can be seen relevant to explain episodes of allegedly spirit possession that manifest with unusual behaviour, trance manifestation, and also the appearance of alters that can take the role of “spiritual beings” speaking through the mouth of the possessed (Perrotta, 2019). Trance states used as a means to communicate with the divine are essential in ecstatic religious and can be seen as acts that have specific community functions essential for the life of the group in which they happen (Padmanabhan, 2017). In this instance, the trance-state is sought by the individual or is pushed by the group to accomplish specific functions (e.g., seeking guidance from spirits) (Lewis, 2002). In other contexts, where illness is seen as the cause of spiritual influence, the ill assumes the connotation of “being possessed” way before experiencing the trance, which is sought afterwards to cure the disease, casting out the evil spirit from the body (Lewis, 2002). Similarly, the perception of FNDs, with the difficulty in finding a physiological nature of the symptoms, can be seen as a supernatural origin, with the illness being of a demonic nature and thus again needing to be exorcised (Kirmayer & Young, 1998). In all these contexts, the personal beliefs and social expectancy dictate how the possession should manifest and how the possessed should behave (Bourguignon, 1976). Manifestation of one or more distinct personalities (i.e., alters) associated with DID can easily fit the description of a possessed individual, who under a specific stressor or ritualistic occurrence can trigger the insurgence of the alter that takes a behaviour in line with the personal beliefs of the individual and the expectancy dictated by the sociocultural context. Similarly, dissociative episodes typical of FNDs can manifest in the allegedly possessed, in response to triggers of various nature, and that are in line with the internal conflict experienced by the individual (Kirmayer & Young, 1998). People reporting negative spirit possession (demonic possession) have often experienced trauma (Hecker et al., 2016). Trance states can be of therapeutic value for the individual experiencing possession. In these rituals, the individual is encouraged to either manifest the “demon” (the culturally influenced alter) or to fall under a culturally dictated hysterical behaviour where the individual is free to express the internal conflict, emotional distress, or trauma (Charles, 1953).

### Exorcising the Mind: The Therapeutic Efficacy of Altered States of Consciousness

The role and significance of ASCs in several cultures have been largely discussed in the literature. When considering the ceremonial and ritualistic procedures that have the function of communicating with the divine, the trance state (divine ecstasy) can be seen as a means to open the door to the spiritual world, allowing spirits to communicate with humans and provide guidance and healing when required (Lewis, 2002). Communication with the spirit can also allow the “possessed” to gain divination powers and dispense prophecy (see the Pythia in ancient Greece) (Johnston, 2008). However, the trance state can also be a way to self-growth, seeking the divine within and becoming one with the Universe, such as in the case of transcendental meditation (Travis & Pearson, 2000). The role of spirit possession can also be seen as a role to provide the oppressed and underrepresented part of a community to actively manifest malcontent and complain about their social situation, leading to a sort of social redemption from the suffering of the unfavourable social status (Lewis, 2002). Personal turmoil and repressed emotion and feelings can be manifested through trance states of symbolic value that provide the stereotyped behaviour dictated by the community to justify the behaviour of the possessed as something out of the control of the possessed (such as in the case of Tarantism or of demonic possessions) (De Martino & Zinn, 2005; Kirmayer & Young, 1998). The externalisation of internal distress on an external agent (the evil spirit, or the tarantula) can be interpreted as a structured framework for the individual to confront and express involuntary or unvoluntary suppressed emotions that cannot be externalised in normal contexts without experiencing moral and social repercussions (De Martino & Zinn, 2005; Kirmayer & Young, 1998). The symbolic nature of the rite induces emotional release and psychological relief, which benefits the individual and allows for their reintegration in the community (Charles, 1953).

In this light, we should look more in detail two distinct roles of the strategies aiming to deal with spirit possession: the exorcism and the adorcism. While the former sees the negative connotation of spirit possession (cause of the mental and physical disease), the latter sees the positive connotation of spirit possession (as guiding spirit who provides healing and guidance) (Bourguignon, 1976). Exorcism is the religious practice aiming to expel a malevolent spiritual entity from an individual or a place. Complexity of the ritual may vary between spiritual practices, but do commonly see the exorcist (the religious leader taking the job of expelling the evil spirit) “commanding” the depart of the malevolent entity (often in the name of a divine authority – e.g., God for the Christians), often interrogating the demon to obtain information on the possession which may help the ritual (e.g., asking for their name, or the reason for possessing the individual) (Charles, 1953; Goodman, 1988). During this process, the possessed individual does enter in a state of induced trance (due to social expectation, religious beliefs, role-play elements based on the religious background, and by the monotonous repetition of prayers by the exorcist), which leads to the manifestation of the other personality (the evil spirit) or to the manifestation of stereotypical behaviour influenced by the evil spirit, which is uncollaborative and aggressive (Goodman, 1988). At the end of the procedure, often the individual receiving the exorcism lacks memory of what happened during the ritual (dissociative amnesia) (Bourguignon, 1976). The exorcism has the function to cast out the evil spirit (the trauma): it is used when the individual sees the possession as a cause of illness and internal turmoil. When strongly rooted in personal and social beliefs, the role of exorcism to cast out the evil spirit may take the connotation of therapy (Goodman, 1988) and should not be disregarded as simple beliefs of a superstitious and unscientific mind. Suppose the individual is truly convinced of being possessed by an external entity, then the exorcism gains therapeutic value, which should be seen and administered in support of other forms of therapeutic interventions (such as medical and psychiatric care, when needed) (Testoni et al., 2025). Approaches such as ego-states therapy (Watkins, 1997) or Gestalt therapy (Perls et al., 1951) have been used in psychotherapy for years as a means to help people manifest repressed emotions and inner turmoil through role-play and parts therapy. Exorcism should be seen to the same extent as these approaches, where the role-playing based on the socio-cognitive model is effective in providing therapeutic help to the patient (Goodman, 1988). The dynamic between the exorcist and the possessed individual sees the exorcist assuming an over-functioning role (taking responsibility for the situation, offering guidance, and exerting control), with the possessed individual assuming the under-functioning role (lacking agency and thus requiring intervention) (Joelsson, 2020). Moreover, in the exorcism rituals, the community assigns the role of authority figure to the exorcist, with the possessed becoming the embodiment of communal fears (role suction) (Horwitz, 1983), externalising the tensions internal at the community, helping them alleviate shared psychological distress.

In contrast to exorcism, adorcism refers to the ritualised incorporation of a spirit or entity with the possessed (De Heusch, 1985). In this context, rather than resisting possession, the individual and the community engage with the spirit (seen as a divine guide) (De Heusch, 1985). Interacting with the spirit is often accomplished through trance, dance, chanting, or other ritual performances (Lewis, 2002). The acceptance of the spirit enables the individual and the community to symbolically process collective tensions, facilitating psychological healing by expressing repressed emotions and conflicts within a safe and structured communal setting (Boddy, 1994). The possessed individual is valorised as a vessel to channel communal fears and to embrace divine guidance to overcome them. In this view, trance and spirit possession contribute to the psychological health of the whole community by fostering their sense of identity, integration, and social belonging, especially in cultures where spirit possession is normalised and ritualised (Boddy, 1994; Lewis, 2002).

A final note on the therapeutic value of ASCs is related to the characteristics of ecstatic dance, observed in spiritual possession rituals such as the ones observed in the Tarantism and in the Haitian Voodoo (Bourguignon, 1976; De Martino & Zinn, 2005). Dancing induces relevant physiological changes in endorphins, serotonin, and dopamine levels (Jeong et al., 2005; Salimpoor et al., 2011; Tarr et al., 2014). These changes promote feelings of euphoria, pain relief, and emotional release, which benefit the individual (Jeong et al., 2005; Salimpoor et al., 2011; Tarr et al., 2014). Moreover, the intense physical exertion experienced during the frenzied dancing, together with the synchronicity of the body movements with the rhythm of drums and chanting, facilitates dissociation (Winkelman, 2010). The release of emotional and physical turmoil while in the dance-induced dissociation facilitates the healing and coping process of the possessed individual (Bourguignon, 1976). Dance and movement are still proposed today in modern forms of transformative practices aiming at mental and physical wellbeing (Koch et al., 2014; López-Rodríguez et al., 2017).

### Considerations for Practice

**Fig. 1:**
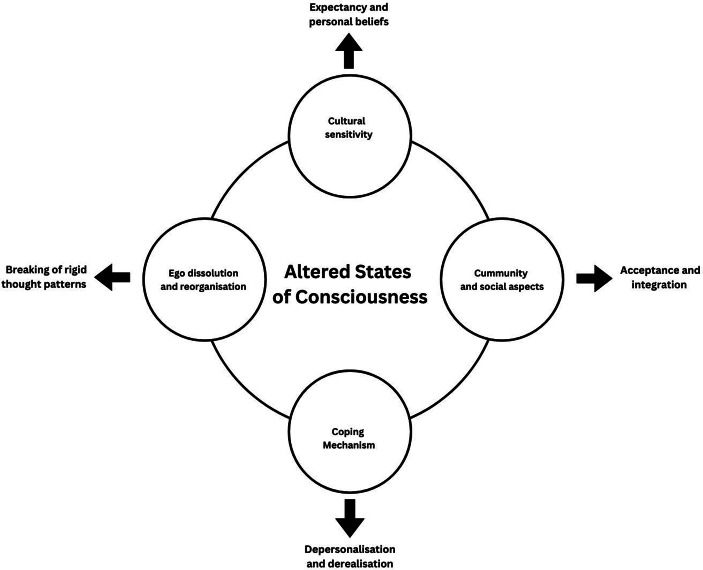
Four major factors in the therapeutic application of altered state of consciousness

Summarising the point discussed so far, therapeutic applications of ASCs have to account for four major factors: (i) cultural sensitivity, (ii) community and social aspects, (iii) ego dissolution and reorganisation, and (iv) coping mechanisms (Fig. 1). The importance of cultural meaning in the use of ASCs is essential to grasp their therapeutic potential. Personal beliefs and social expectancy are fundamental in establishing a proper patient-therapist connection (Lynn & Rhue, 1991), and understanding the cultural frame from which these beliefs come is something that needs to be evaluated in depth when assessing and planning interventions for mental health. It has been largely argued how Psychiatry tends towards a model where cultural differences are often ignored in the diagnosis of mental conditions (Alarcòn, 2009; Bredström, 2019), without a proper inclusive model that accounts for sociocultural perception of specific mental states seen as normal and healthy when part of a context, while unhealthy in others. Similarly, techniques to induce ASCs, such as focused meditation or Japa meditation (Srinivasan, 2013) cannot be simply deprived of their cultural roots. While the West has tried to integrate Eastern meditative practices in therapeutic contexts (e.g., mindfulness), it is also true that during the process, these practices have been largely deprived of their cultural meaning, often leading to methods that do not often obtain the expected results. Additionally, it is important to note how procedures to induce ASCs have always used indigenous knowledge, such as fasting and intense physical exhaustion to induce ASCs (Anālayo, 2021), which despite they cannot fit nowadays therapeutic standards, it is also true that their removal may lead to incomplete procedures that lose or reduce their ability to induce the desired results. Community and relationship aspects play an important role in making the experience immersive and allow the individual to express their inner turmoil. Again, looking at the case of spirit possession, community rules and roles can be subverted in regulated and accepted rituals that help manifest discontent and internal turmoil without facing harsh consequences (e.g., social judgment) (Lewis, 2002).

Therapeutic value of ASCs also come from ego dissolution (i.e., the temporary reduction or loss of one’s sense of self or identity) which occurs due to a disruption in the brain’s self-modelling processes (which are primarily supported by the DMN) (Letheby & Gerrans, 2017) and that helps in emotional release and catharsis, as well as in breaking rigid thought patterns and thus facilitating psychological flexibility and behavioural changes. ASCs also work as a coping mechanism, with dissociation known to be triggered during traumatic events (DePrince and Freyd, 2007), helping the person to disconnect from intense emotional and physical pain. The same mechanism of disconnection from one’s own emotion can also be relevant in suppressing unwanted thoughts and reducing emotional reactivity (Oathes & Ray, 2008).

## Conclusions

Over the centuries, ASCs have been integrated into complex rituals, assuming cultural meaning dependent on the social context. The cultural value of ASCs has to be considered a fundamental aspect of the experience of trance-like states, as its sociocultural component is essential to shape the emotional weight of the experience. Spirit possession, as an example of cultural integration of ASCs in spiritual and religious practices, clearly demonstrates how ASCs can assume different meanings and values based on the community of reference. In the same way, the expression of trauma, emotional distress, and internal turmoil has been regulated by sociocultural norms through the ritualistic use of ASCs, making trance-like states a powerful tool to channelling fears and conflicts of the community, leading to their regulated resolution. The sociocultural component of ASCs is relevant not only on an anthropological or psychological level, but also for a clinical understanding of states of dissociation. Cultural influences strongly dictate the perception of mental states, and dissociation and ASCs cannot be torn apart from their sociocultural elements. Social expectancy and personal beliefs dictate the effectiveness of therapeutic interventions that use ASCs (e.g., hypnosis, mindfulness), making it essential to grasp the cultural and symbolic value these have for the individual. Although understanding the neurophysiological mechanisms of ASCs is an essential part of the study of consciousness, the cultural meaning associated with trance-like states needs to be considered as of the same importance to gain a proper understanding of the whole aspect of human consciousness.

## Data Availability

No datasets were generated or analysed during the current study.

## References

[CR1] Alarcòn, R. D. (2009). Culture, cultural factors and psychiatric diagnosis: Review and projections. *World Psychiatry*, *8*(3), 131. 19812742 10.1002/j.2051-5545.2009.tb00233.x PMC2755270

[CR2] American Psychiatric Association (2022). *Diagnostic and statistical manual of mental disorders (5th ed., text rev.; DSM-5-TR)*. American Psychiatric Publishing.

[CR3] Anālayo, B. (2021). The buddha’s pre-awakening practices and their mindful transformation. *Mindfulness*, *12*(8), 1892–1898.

[CR4] Andrews-Hanna, J. R., Smallwood, J., & Spreng, R. N. (2014). The default network and self-generated thought: Component processes, dynamic control, and clinical relevance. *Annals of the New York Academy of Sciences*, *1316*(1), 29–52. 10.1111/nyas.12360 24502540 10.1111/nyas.12360 PMC4039623

[CR5] Ano, G. G., & Vasconcelles, E. B. (2005). Religious coping and psychological adjustment to stress: A meta-analysis. *Journal of Clinical Psychology*, *61*(4), 461–480. 10.1002/jclp.20049 15503316 10.1002/jclp.20049

[CR6] Aybek, S., & Perez, D. L. (2022). Diagnosis and management of functional neurological disorder. *Bmj 376*. 10.1136/bmj.o64 10.1136/bmj.o64 35074803

[CR7] Aybek, S., & Vuilleumier, P. (2016). Chapter 7 - Imaging studies of functional neurologic disorders. In M. Hallett, J. Stone, & A. Carson (Eds.), *Handbook of Clinical Neurology* (Vol. 139, pp. 73–84). Elsevier. 10.1016/B978-0-12-801772-2.00007-2 10.1016/B978-0-12-801772-2.00007-2 27719879

[CR8] Belli, H., Akbudak, M., Ural, C., Solmaz, M., Dogan, Z., & Konkan, R. (2017). Is there a complex relation between social anxiety disorder, childhood traumatic experiences and dissociation? *Nordic Journal of Psychiatry*, *71*(1), 55–60. 10.1080/08039488.2016.1218050 27564540 10.1080/08039488.2016.1218050

[CR9] Boddy, J. (1994). Spirit possession revisited: Beyond instrumentality. *Annual Review of Anthropology*, 407–434.

[CR10] Bonnelle, V., Ham, T. E., Leech, R., Kinnunen, K. M., Mehta, M. A., Greenwood, R. J., & Sharp, D. J. (2012). Salience network integrity predicts default mode network function after traumatic brain injury. *Proceedings of the National Academy of Sciences*, *109*(12), 4690–4695. 10.1073/pnas.1113455109 10.1073/pnas.1113455109 PMC3311356 22393019

[CR11] Bourguignon, E. (1976). Possession.

[CR12] Bozhilova, N. S., Michelini, G., Kuntsi, J., & Asherson, P. (2018). Mind wandering perspective on attention-deficit/hyperactivity disorder. *Neuroscience & Biobehavioral Reviews*, *92*, 464–476. 10.1016/j.neubiorev.2018.07.010 30036553 10.1016/j.neubiorev.2018.07.010 PMC6525148

[CR13] Bredström, A. (2019). Culture and context in mental health diagnosing: Scrutinizing the DSM-5 revision. *Journal of Medical Humanities*, *40*(3), 347–363. 29282590 10.1007/s10912-017-9501-1 PMC6677698

[CR14] Butler, L. D. (2006). Normative dissociation. *Psychiatric Clinics*, *29*(1), 45–62. 10.1016/j.psc.2005.10.004 16530586 10.1016/j.psc.2005.10.004

[CR15] Canna, M., & Seligman, R. (2020). Dealing with the unknown. Functional neurological disorder (FND) and the conversion of cultural meaning. *Social Science & Medicine*, *246*, 112725. 10.1016/j.socscimed.2019.112725 31911360 10.1016/j.socscimed.2019.112725

[CR16] Cavanna, A., Cavanna, S., & Cavanna, A. (2010). Epileptic seizures and spirit possession in Haitian culture: Report of four cases and review of the literature. *Epilepsy & Behavior*, *19*(1), 89–91. 10.1016/j.yebeh.2010.07.002 20724222 10.1016/j.yebeh.2010.07.002

[CR17] Chambers, A. C. (2021). Somewhere between science and superstition’: Religious outrage, horrific science, and the exorcist (1973). *History of the Human Sciences*, *34*(5), 32–52. 10.1177/09526951211004465 34776653 10.1177/09526951211004465 PMC8575976

[CR18] Charles, L. H. (1953). Drama in Shaman exorcism. *The Journal of American Folklore*, *66*(260), 95–122.

[CR19] Cleary, S. (2022). Better the devil you know: The myth of harm and the satanic panic. *Gothic Studies*, *24*(2), 167–184. 10.3366/gothic.2022.0132

[CR20] Cohen, E. (2008). What is spirit possession? Defining, comparing, and explaining two possession forms. *Ethnos*, *73*(1), 101–126. 10.1080/00141840801927558

[CR21] Cook, C. C. (2025). Demon possession, theology, and mental health. *Journal of Disability & Religion*, *29*(2), 171–189. 10.1080/23312521.2024.2441435

[CR22] Cook, C. C., & Hamley, I. (2020). *The bible and mental health: Towards a biblical theology of mental health*. SCM.

[CR23] Coutinho, J. F., Fernandesl, S. V., Soares, J. M., Maia, L., Gonçalves Ó, F., & Sampaio, A. (2016). Default mode network dissociation in depressive and anxiety States. *Brain Imaging Behav*, *10*(1), 147–157. 10.1007/s11682-015-9375-7 25804311 10.1007/s11682-015-9375-7

[CR24] De Heusch, L. (1985). *Sacrifice in africa: A structuralist approach*. Manchester University.

[CR25] De Martino, E., & Zinn, D. L. (2005). *The land of remorse: A study of Southern Italian tarantism*. Free Association.

[CR26] Deeley, Q., Oakley, D. A., Toone, B., Giampietro, V., Brammer, M. J., Williams, S. C., & Halligan, P. W. (2012). Modulating the default mode network using hypnosis. *International Journal of Clinical and Experimental Hypnosis*, *60*(2), 206–228. 10.1080/00207144.2012.648070 22443526 10.1080/00207144.2012.648070

[CR277] DePrince, A. P., & Freyd, J. J. (2007). Trauma-induced dissociation. In M. J. Friedman, T. M. Keane, & P. A. Resick (Eds.), Handbook of PTSD: *Science and practice* (pp. 135–150). The Guilford Press. https://psycnet.apa.org/record/2007-14029-008

[CR27] Desmangles, L. G. (2000). *The faces of the gods: Vodou and Roman Catholicism in Haiti*. Univ of North Carolina.

[CR28] Espay, A. J., Aybek, S., Carson, A., Edwards, M. J., Goldstein, L. H., Hallett, M., LaFaver, K., LaFrance, W. C., Lang, A. E., & Nicholson, T. (2018). Current concepts in diagnosis and treatment of functional neurological disorders. *JAMA Neurology*, *75*(9), 1132–1141. 10.1001/jamaneurol.2018.1264 29868890 10.1001/jamaneurol.2018.1264 PMC7293766

[CR29] Feane, K., & Uprichard, J. (2025). Management of patients who refuse blood transfusion. *Medicine*. 10.4103/0019-5049.144680

[CR30] Ficek-Tani, B., Horien, C., Ju, S., Xu, W., Li, N., Lacadie, C., Shen, X., Scheinost, D., Constable, T., & Fredericks, C. (2023). Sex differences in default mode network connectivity in healthy aging adults. *Cerebral Cortex*, *33*(10), 6139–6151. 10.1093/cercor/bhac491 36563018 10.1093/cercor/bhac491 PMC10183749

[CR31] Freud, S. (2012). *Totem and taboo*. Routledge.

[CR32] Giordan, G., & Possamai, A. (2016). The over-policing of the devil: A sociology of exorcism. *Social Compass*, *63*(4), 444–460. 10.1177/0037768616663982

[CR33] Goodman, F. D. (1988). *How about demons? Possession and exorcism in the modern world*. Indiana University Press.

[CR34] Goulden, N., Khusnulina, A., Davis, N. J., Bracewell, R. M., Bokde, A. L., McNulty, J. P., & Mullins, P. G. (2014). The salience network is responsible for switching between the default mode network and the central executive network: Replication from DCM. *Neuroimage*, *99*, 180–190. 10.1016/j.neuroimage.2014.05.052 24862074 10.1016/j.neuroimage.2014.05.052

[CR35] Hallett, M., Aybek, S., Dworetzky, B. A., McWhirter, L., Staab, J. P., & Stone, J. (2022). Functional neurological disorder: New subtypes and shared mechanisms. *The Lancet Neurology*, *21*(6), 537–550. 10.1016/S1474-4422(21)00422-1 35430029 10.1016/S1474-4422(21)00422-1 PMC9107510

[CR36] Hecker, T., Barnewitz, E., Stenmark, H., & Iversen, V. (2016). Pathological spirit possession as a cultural interpretation of trauma-related symptoms. *Psychological Trauma: Theory Research Practice and Policy*, *8*(4), 468. 10.1037/tra0000117 27031081 10.1037/tra0000117

[CR37] Heeßel, N. P. (2004). Diagnosis, divination and disease: towards an understanding of the rationale behind the Babylonian Diagnostic Handbook. In *Magic and rationality in ancient near Eastern and Graeco-Roman medicine* (pp. 97–116). Brill. 17152169

[CR39] Horwitz, L. (1983). Projective identification in dyads and groups. *International Journal of Group Psychotherapy*, *33*(3), 259–279. 6885211 10.1080/00207284.1983.11490877

[CR40] Jacques Jouanna, B., & Van der Eijk, P. (2012). *Greek medicine from Hippocrates to galen: Selected papers*. Brill.

[CR41] James, W. (1902). *The varieties of religious experience: A study in human nature; being the gifford lectures on natural religion delivered at Edinburgh in 1901–1902*. Longmans.

[CR42] Jeong, Y. J., Hong, S. C., Lee, M. S., Park, M. C., Kim, Y. K., & Suh, C. M. (2005). Dance movement therapy improves emotional responses and modulates neurohormones in adolescents with mild depression. *International Journal of Neuroscience*, *115*(12), 1711–1720. 10.1080/00207450590958574 16287635 10.1080/00207450590958574

[CR43] Jiang, H., White, M. P., Greicius, M. D., Waelde, L. C., & Spiegel, D. (2017). Brain activity and functional connectivity associated with hypnosis. *Cerebral Cortex*, *27*(8), 4083–4093. 10.1093/cercor/bhw220 27469596 10.1093/cercor/bhw220 PMC6248753

[CR44] Joelsson, L. (2020). Exorcisms as liberation: Trauma, differentiation, and social systems in Luke. *Studia Theologica-Nordic Journal of Theology*, *74*(2), 159–196.

[CR45] Johnston, S. I. (2008). *Ancient Greek divination*. Wiley-Blackwell.

[CR46] Josephus, F. (2006). *Jewish antiquities*. Wordsworth Editions.

[CR47] Karenberg, A., & Leitz, C. (2001). Headache in magical and medical papyri of ancient Egypt. *Cephalalgia*, *21*(9), 911–916. 10.1046/j.1468-2982.2001.00274.x 11903286 10.1046/j.1468-2982.2001.00274.x

[CR48] Kate, N., Grover, S., Kulhara, P., & Nehra, R. (2012). Supernatural beliefs, aetiological models and help seeking behaviour in patients with schizophrenia. *Industrial Psychiatry Journal*, *21*(1), 49–54. 10.4103/0972-6748.110951 23766578 10.4103/0972-6748.110951 PMC3678179

[CR49] Kawamura, K. (2003). A female shaman’s mind and body, and possession. *Asian Folklore Studies*, 257–289.

[CR50] Kihlstrom, J. F., & Evans, F. J. (2014). Memory retrieval processes during posthypnotic amnesia. *Functional disorders of memory (PLE: memory)* (pp. 179–218). Psychology.

[CR51] Kirmayer, L. J. (2001). Cultural variations in the clinical presentation of depression and anxiety: Implications for diagnosis and treatment. *Journal of Clinical Psychiatry*, *62*, 22–30. 11434415

[CR52] Kirmayer, L. J., & Santhanam, R. (2000). The anthropology of hysteria. In P. W. Halligan, C. Bass, & J. C. Marshall (Eds.), *Contemporary approaches to the study of hysteria: Clinical and theoretical perspectives* (pp. 251–270). Oxford University Press.

[CR53] Kirmayer, L. J., & Young, A. (1998). Culture and somatization: Clinical, epidemiological, and ethnographic perspectives. *Psychosomatic Medicine*, *60*(4), 420–430. 10.1097/00006842-199807000-00006 9710287 10.1097/00006842-199807000-00006

[CR54] Koch, S., Kunz, T., Lykou, S., & Cruz, R. (2014). Effects of dance movement therapy and dance on health-related psychological outcomes: A meta-analysis. *The Arts in Psychotherapy*, *41*(1), 46–64. 10.3389/fpsyg.2019.01806eCollection 2019.

[CR55] Krause-Utz, A., Frost, R., Winter, D., & Elzinga, B. M. (2017). Dissociation and alterations in brain function and structure: Implications for borderline personality disorder. *Current Psychiatry Reports*, *19*, 1–22. 10.1007/s11920-017-0757-y 28138924 10.1007/s11920-017-0757-y PMC5283511

[CR56] Lanius, R. A. (2015). Trauma-related dissociation and altered States of consciousness: A call for clinical, treatment, and neuroscience research. *Eur J Psychotraumatol*, *6*, 27905. 10.3402/ejpt.v6.27905 25994026 10.3402/ejpt.v6.27905 PMC4439425

[CR57] Leal, M., Bezerra, E., & de Freitas, M. (2024). Role of belief in miracles in clinical settings-a literature review. *J Psychol Clin Psychiatry*, *10*. 10.15406/jpcpy.2024.15.00756

[CR58] Lebois, L. A., Kumar, P., Palermo, C. A., Lambros, A. M., O’Connor, L., Wolff, J. D., Baker, J. T., Gruber, S. A., Lewis-Schroeder, N., & Ressler, K. J. (2022). Deconstructing dissociation: A triple network model of trauma-related dissociation and its subtypes. *Neuropsychopharmacology: official Publication of the American College of Neuropsychopharmacology*, *47*(13), 2261–2270. 10.1038/s41386-022-01468-1 36202907 10.1038/s41386-022-01468-1 PMC9630268

[CR59] Leo, D. G., Keller, S. S., & Proietti, R. (2024). Close your eyes and relax: The role of hypnosis in reducing anxiety, and its implications for The prevention of cardiovascular diseases. *Frontiers in Psychology*, *15*, 1411835. 10.3389/fpsyg.2024.1411835 39035095 10.3389/fpsyg.2024.1411835 PMC11258040

[CR60] Letheby, C., & Gerrans, P. (2017). Self unbound: Ego dissolution in psychedelic experience. *Neuroscience of Consciousness*, *2017*(1), nix016. 30042848 10.1093/nc/nix016 PMC6007152

[CR61] Lewis, I. M. (2002). *Ecstatic religion: A study of shamanism and spirit possession*. Routledge.

[CR62] Lloyd, C. E., & Panagopoulos, M. C. (2022). Mad, bad, or possessed’? Perceptions of self-harm and mental illness in evangelical Christian communities. *Pastoral Psychology*, *71*(3), 291–311.

[CR63] Lloyd, C. E. M., & Panagopoulos, M. C. (2023). Narratives of externality, oppression, and agency: Perceptions of the role of the demonic in mental illness among evangelical Christians. *Pastoral Psychology*, *72*(4), 501–523. 10.1007/s11089-023-01079-7

[CR64] Lofthouse, M. K., Waite, P., & Černis, E. (2023). Developing an Understanding of the relationship between anxiety and dissociation in adolescence. *Psychiatry Research*, *324*, 115219. 10.1016/j.psychres.2023.115219 37119790 10.1016/j.psychres.2023.115219

[CR65] López-Rodríguez, M. M., Baldrich-Rodríguez, I., Ruiz-Muelle, A., Cortés-Rodríguez, A. E., Lopezosa-Estepa, T., & Roman, P. (2017). Effects of Biodanza on stress, depression, and sleep quality in university students. *Journal of Alternative and Complementary Medicine*, *23*(7), 558–565. 10.1089/acm.2016.0365 28590767 10.1089/acm.2016.0365

[CR66] Luhrmann, T. M., Padmavati, R., Tharoor, H., & Osei, A. (2015). Hearing voices in different cultures: A social kindling hypothesis. *Topics in Cognitive Science*, *7*(4), 646–663. 10.1111/tops.12158 26349837 10.1111/tops.12158

[CR67] Lutz, A., Dunne, J. D., & Davidson, R. J. (2006). Meditation and the neuroscience of consciousness: An introduction. *The Cambridge handbook of consciousness*, *19*.

[CR68] Lynn, S. J., & Rhue, J. W. (1991). An integrative model of hypnosis. *Theories of Hypnosis: Current Models and Perspectives*, 397–438.

[CR69] Mak, L. E., Minuzzi, L., MacQueen, G., Hall, G., Kennedy, S. H., & Milev, R. (2017). The default mode network in healthy individuals: A systematic review and meta-analysis. *Brain Connectivity*, *7*(1), 25–33. 10.1089/brain.2016.0438 27917679 10.1089/brain.2016.0438

[CR70] Marie, N., Lafon, Y., Bicego, A., Grégoire, C., Rousseaux, F., Bioy, A., Vanhaudenhuyse, A., & Gosseries, O. (2024). Scoping review on shamanistic trances practices. *BMC Complementary Medicine and Therapies*, *24*(1), 381. 39497104 10.1186/s12906-024-04678-w PMC11536825

[CR71] Mathison, L. A., Jackson, R., & Wade, N. G. (1895). Stigma and Mental Health in the Abrahamic Religious Traditions. *communities*, *2004*, 34. In: Vogel DL, Wade NG, eds. *The Cambridge Handbook of Stigma and Mental Health*. Cambridge Handbooks in Psychology. Cambridge University Press; 2022:347–366.

[CR72] Menon, V. (2011). Large-scale brain networks and psychopathology: A unifying triple network model. *Trends in Cognitive Sciences*, *15*(10), 483–506. 10.1016/j.tics.2011.08.003 21908230 10.1016/j.tics.2011.08.003

[CR73] Merckelbach, H., Muris, P., & Rassin, E. (1999). Fantasy proneness and cognitive failures as correlates of dissociative experiences. *Personality and Individual Differences*, *26*(5), 961–967. 10.1016/S0191-8869(98)00193-7

[CR74] Monsa, R., Peer, M., & Arzy, S. (2018). Self-reference, emotion Inhibition and somatosensory disturbance: Preliminary investigation of network perturbations in conversion disorder. *European Journal of Neurology*, *25*(6), 888–e862. 10.1111/ene.13613 29509290 10.1111/ene.13613

[CR75] Morton, J. (2017). Interidentity amnesia in dissociative identity disorder. *Cognitive Neuropsychiatry*, *22*(4), 315–330. 10.1080/13546805.2017.1327848 28545341 10.1080/13546805.2017.1327848

[CR76] Nooney, J., & Woodrum, E. (2002). Religious coping and church-based social support as predictors of mental health outcomes: Testing a conceptual model. *Journal for the Scientific Study of Religion*, *41*(2), 359–368. 10.1111/1468-5906.00122

[CR77] Nutton, V. (2012). *Ancient medicine*. Routledge.

[CR78] Oathes, D. J., & Ray, W. J. (2008). Dissociative tendencies and facilitated emotional processing. *Emotion*, *8*(5), 653. 18837615 10.1037/a0013442 PMC2683754

[CR79] Padmanabhan, D. (2017). From distress to disease: A critique of the medicalisation of possession in DSM-5. *Anthropology & Medicine*, *24*(3), 261–275. 10.1080/13648470.2017.1389168 29283036 10.1080/13648470.2017.1389168

[CR80] Page, R. A., & Green, J. P. (2007). An update on age, hypnotic suggestibility, and gender: A brief report. *American Journal of Clinical Hypnosis*, *49*(4), 283–287. 10.1080/00029157.2007.10524505 17444365 10.1080/00029157.2007.10524505

[CR81] Pargament, K. I. (2001). *The psychology of religion and coping: Theory, research, practice*. Guilford Press.

[CR82] Pekala, R. J., Kumar, V., Maurer, R., Elliott-Carter, N., Moon, E., & Mullen, K. (2010). Suggestibility, expectancy, trance state effects, and hypnotic depth: I. Implications for Understanding hypnotism. *American Journal of Clinical Hypnosis*, *52*(4), 275–290. 10.1080/00029157.2010.10401732 20499542 10.1080/00029157.2010.10401732

[CR83] Perls, F., Hefferline, G., & Goodman, P. (1951). Gestalt therapy. *New York*, *64*(7), 19–313.

[CR84] Perrotta, G. (2019). The phenomenon of demonic possession: Definition, contexts and multidisciplinary approaches. *J Psychology and Mental Health Care*. 10.31579/2637-8892/019

[CR85] Peter, B. (2005). Gassner’s exorcism—not mesmer’s magnetism—is the real predecessor of modern hypnosis. *International Journal of Clinical and Experimental Hypnosis*, *53*(1), 1–12. 10.1080/00207140490914207 15788240 10.1080/00207140490914207

[CR86] Pietkiewicz, I. J., Kłosińska, U., & Tomalski, R. (2021). Delusions of possession and religious coping in schizophrenia: A qualitative study of four cases. *Frontiers in Psychology*, *12*, 628925. 10.3389/fpsyg.2021.628925 33815215 10.3389/fpsyg.2021.628925 PMC8017190

[CR87] Preller, K. H., & Vollenweider, F. X. (2018). Phenomenology, structure, and dynamic of psychedelic States. *Behavioral Neurobiology of Psychedelic Drugs*, 221–256. 10.1007/7854_2016_459 10.1007/7854_2016_459 28025814

[CR88] Price-Williams, D., & Hughes, D. J. (1994). Shamanism and altered States of consciousness. *Anthropology of Consciousness*, *5*(2), 1–15. 10.1525/ac.1994.5.2.1

[CR89] Randal, P., Geekie, J., Lambrecht, I., & Taitimu, M. (2018). Dissociation, psychosis and spirituality: Whose voices are we hearing? *Psychosis Trauma and Dissociation: Evolving Perspectives on Severe Psychopathology*, *427–439*. 10.1002/9780470699652.ch24

[CR90] Raynor, G., & Baslet, G. (2019). Functional neurological disorder and dissociative disorders in women. *Neurology and Psychiatry of Women: A Guide to Gender-based Issues in Evaluation, Diagnosis, and Treatment*, 15–26. 10.1007/978-3-030-04245-5_3

[CR91] Retief, F., & Cilliers, L. (2007). Mesopotamian medicine. *SAMJ*, *97*(1), 27–29. 17378276

[CR92] Robbins, J. (2004). The globalization of pentecostal and charismatic Christianity. *Annual Review of Anthropology*, *33*(1), 117–143. 10.1146/annurev.anthro.32.061002.093421

[CR93] Russell, J. B. (1987). *The devil: Perceptions of evil from antiquity to primitive Christianity*. Cornell University Press.

[CR94] Salimpoor, V. N., Benovoy, M., Larcher, K., Dagher, A., & Zatorre, R. J. (2011). Anatomically distinct dopamine release during anticipation and experience of peak emotion to music. *Nature Neuroscience*, *14*(2), 257–262. 10.1038/nn.2726 21217764 10.1038/nn.2726

[CR95] Savel, R. H., & Munro, C. L. (2014). *From asclepius to hippocrates: The Art and science of healing* (Vol. 23, pp. 437–439). American Association of Critical Care Nurses. 10.4037/ajcc2014993 25362664

[CR96] Seligman, R., & Kirmayer, L. J. (2008). Dissociative experience and cultural neuroscience: Narrative, metaphor and mechanism. *Culture Medicine and Psychiatry*, *32*, 31–64. 10.1007/s11013-007-9077-8 18213511 10.1007/s11013-007-9077-8 PMC5156567

[CR98] Shen, K. K., Welton, T., Lyon, M., McCorkindale, A. N., Sutherland, G. T., Burnham, S., Fripp, J., Martins, R., & Grieve, S. M. (2020). Structural core of the executive control network: A high angular resolution diffusion MRI study. *Human Brain Mapping*, *41*(5), 1226–1236. 10.1002/hbm.24870 31765057 10.1002/hbm.24870 PMC7267982

[CR99] Simba, H., Mmbaga, B. T., Serventi, F., Mremi, A., Motlhale, M., Espina, C., Mwasamwaja, A., Schuz, J., McCormack, V., & Prah, E. (2023). Why am I ill? Beliefs in supernatural and natural causes of ill? health at the time of diagnostic workup of patients with esophageal cancer in Tanzania. *JCO Global Oncology*, *9*, e2300100. 10.1200/GO.23.00100 37883724 10.1200/GO.23.00100 PMC10846787

[CR100] Spiegel, D., Loewenstein, R. J., Lewis-Fernández, R., Sar, V., Simeon, D., Vermetten, E., Cardeña, E., & Dell, P. F. (2011). Dissociative disorders in DSM‐5. *Depression and Anxiety*, *28*(9), 824–852. 10.1002/da.20874 21910187 10.1002/da.20874

[CR101] Srinivasan, T. M. (2013). From meditation to Dhyana. *International Journal of Yoga*, *6*(1), 1–3. 23436967 10.4103/0973-6131.105934 PMC3573536

[CR102] Staniloiu, A., & Markowitsch, H. J. (2014). Dissociative amnesia. *The Lancet Psychiatry*, *1*(3), 226–241. 10.1016/S2215-0366(14)70279-2 26360734 10.1016/S2215-0366(14)70279-2

[CR103] Taïb, S., Yrondi, A., Lemesle, B., Péran, P., & Pariente, J. (2023). What are the neural correlates of dissociative amnesia? A systematic review of the functional neuroimaging literature. *Frontiers in Psychiatry*, *14*, 1092826. 10.3389/fpsyt.2023.1092826 36778638 10.3389/fpsyt.2023.1092826 PMC9909275

[CR104] Tarr, B., Launay, J., & Dunbar, R. I. (2014). Music and social bonding:self-other merging and neurohormonal mechanisms. *Frontiers in Psychology*, *5*, 1096. 10.3389/fpsyg.2014.01096 25324805 10.3389/fpsyg.2014.01096 PMC4179700

[CR105] Testoni, I., Vischio, A., De Bona, D., Gentile, M., & De Vincenzo, C. (2025). Believing in possession: Social psychological distress and the need for spiritually oriented psychological and pastoral support. *Pastoral Psychology*, 1–17. 10.1007/s11089-025-01217-3

[CR38] *The Holy Bible: New King James Version.* (1982). Thomas Nelson.

[CR106] Tiwana, M. H., & Smith, J. (2024). Faith and vaccination: A scoping review of the relationships between religious beliefs and vaccine hesitancy. *Bmc Public Health*, *24*(1), 1806. 10.1186/s12889-024-18873-4 38971784 10.1186/s12889-024-18873-4 PMC11227154

[CR107] Travis, F., & Pearson, C. (2000). Pure consciousness: Distinct phenomenological and physiological correlates of consciousness itself. *International Journal of Neuroscience*, *100*(1–4), 77–89. 10.3109/00207450008999678 10512549

[CR108] Underhill, E. (2002). *Mysticism: A study in the nature and development of spiritual consciousness*. Courier Corporation.

[CR109] Vaitl, D., Birbaumer, N., Gruzelier, J., Jamieson, G. A., Kotchoubey, B., Kübler, A., Strehl, U., Lehmann, D., Miltner, W. H., & Weiss, T. (2013). Psychobiology of altered states of consciousness. 10.1037/0033-2909.131.1.98 10.1037/0033-2909.131.1.98 15631555

[CR110] Vermetten, E., Schmahl, C., Lindner, S., Loewenstein, R. J., & Bremner, J. D. (2006). Hippocampal and amygdalar volumes in dissociative identity disorder. *American Journal of Psychiatry*, *163*(4), 630–636. 10.1176/ajp.2006.163.4.630 16585437 10.1176/appi.ajp.163.4.630 PMC3233754

[CR111] Walker, J. (1990). The place of magic in the practice of medicine in ancient Egypt. *Macquarie University Journal Contribution*. https://hdl.handle.net/1959.14/26392630.v1

[CR112] Ward, C. (1980). Spirit possession and mental health: A psycho-anthropological perspective. *Human Relations*, *33*(3), 149–163. 10.1177/001872678003300301

[CR113] Watkins, J. G., & Watkins, H. H. (1997). *Ego states: Theory and therapy*. W. W. Norton & Company.

[CR114] Wieder, L., Brown, R. J., Thompson, T., & Terhune, D. B. (2022). Hypnotic suggestibility in dissociative and related disorders: A meta-analysis. *Neuroscience & Biobehavioral Reviews*, *139*, 104751. 10.1016/j.neubiorev.2022.104751 35760389 10.1016/j.neubiorev.2022.104751

[CR115] Winkelman, M. (2010). Shamanism: A biopsychosocial paradigm of consciousness and healing. *Bloomsbury Publishing*. 10.5040/9798216014133. 2nd ed.

[CR116] Wolfradt, U. (1997). Dissociative experiences, trait anxiety and paranormal beliefs. *Personality and Individual Differences*, *23*(1), 15–19. 10.1016/S0191-8869(97)00043-3

[CR117] World Health Organization, W. H. O (2019). *6B63 Possession trance disorder*. Retrieved 13 May from https://icd.who.int/browse/2025-01/mms/en#1374925579

[CR118] Young, F. (2016). *A history of exorcism in Catholic Christianity*. Springer. 10.1007/978-3-319-29112-3

[CR119] Yuan, M., Liu, B., Yang, B., Dang, W., Xie, H., Lui, S., Qiu, C., Zhu, H., & Zhang, W. (2023). Dysfunction of default mode network characterizes generalized anxiety disorder relative to social anxiety disorder and post-traumatic stress disorder. *Journal of Affective Disorders*, *334*, 35–42. 10.1016/j.jad.2023.04.099 37127115 10.1016/j.jad.2023.04.099

[CR120] Zhou, X., & Lei, X. (2018). Wandering Minds with wandering brain networks. *Neuroscience Bulletin*, *34*(6), 1017–1028. 10.1007/s12264-018-0278-7 30136075 10.1007/s12264-018-0278-7 PMC6246840

[CR121] Zucconi, L. M. (2007). Medicine and religion in ancient Egypt. *Religion Compass*, *1*(1), 26–37. 10.1111/j.1749-8171.2006.00004.x

